# Concordance of Genomic Alterations between Circulating Tumor DNA and Matched Tumor Tissue in Chinese Patients with Breast Cancer

**DOI:** 10.1155/2020/4259293

**Published:** 2020-08-27

**Authors:** Bing Xu, Guangyu Shan, Qixi Wu, Weiwei Li, Hongjiang Wang, Hui Li, Yaping Yang, Qiming Long, Ping Zhao

**Affiliations:** ^1^Bioinformatics Department, Beijing USCI Medical Laboratory, No. 7, Zone C, Yiyuan Cultural Innovation Base, No. 65 Xingshikou Road, Haidian District, Beijing 100195, China; ^2^Research and Development Department, Beijing USCI Medical Laboratory, No. 7, Zone C, Yiyuan Cultural Innovation Base, No. 65 Xingshikou Road, Haidian District, Beijing 100195, China; ^3^Department of Breast Surgery, First Affiliated Hospital of Dalian Medical University, Zhongshan Road 222, Dalian 116011, China; ^4^Department of Breast Surgery, Sichuan Cancer Hospital, Chengdu 610041, China; ^5^Guangdong Provincial Key Laboratory of Malignant Tumor Epigenetics and Gene Regulation, Breast Tumor Center, Sun Yat-sen Memorial Hospital, Sun Yat-sen University, Guangzhou, Guangdong 510120, China; ^6^Cancer Foundation of China Office, 10th Floor, Building 2, Guangqu Home, Guangqumen, Dongcheng District, Beijing 100061, China

## Abstract

**Purpose:**

Circulating tumor DNA (ctDNA) served as a noninvasive method with less side effects using peripheral blood. Given the studies on concordance rate between liquid and solid biopsies in Chinese breast cancer (BC) patients were limited, we sought to examine the concordance rate of different kinds of genomic alterations between paired tissue biopsies and ctDNA samples in Chinese BC cohorts.

**Materials and Methods:**

In this study, we analyzed the genomic alteration profiles of 81 solid BC samples and 41 liquid BC samples. The concordance across 136 genes was evaluated.

**Results:**

The median mutation counts per sample in 41 ctDNA samples was higher than the median in 81 tissue samples (*p*=0.0254; Wilcoxon rank sum test). For mutation at the protein-coding level, 39.0% (16/41) samples had at least one concordant mutation in two biopsies. 20.0% tissue-derived mutations could be detected via ctDNA-based sequencing, whereas 11.7% ctDNA-derived mutations could be found in paired tissues. At gene amplification level, the overall concordant rate was 68.3% (28/41). The concordant rate at gene level for each patient ranged from 83.8% (114/136) to 99.3% (135/136). And, the mean level of variant allele frequency (VAF) for concordant mutations in ctDNA was statistically higher than that for the discordant ones (*p* < 0.001; Wilcoxon rank sum test). Across five representative genes, the overall sensitivity and specificity were 49.0% and 85.9%, respectively.

**Conclusion:**

Our results indicated that ctDNA could provide complementary information on genetic characterizations in detecting single nucleotide variants (SNVs) and insertions and deletions (InDels).

## 1. Introduction

Increasing applications of Next-Generation Sequencing (NGS) in oncology have helped scientists and clinicians in depicting the genomic landscape of breast cancer (BC) [1–3]. NGS technology has been applied to analyze genomic alterations in tumor tissue and more recently in circulating tumor DNA (ctDNA) in clinical testing. Tissue biopsy has been regarded as a gold standard for molecular profiling of solid tumor but has several limitations in practical use. It can be invasive with a higher possibility to cause side effects compared with liquid biopsy [4, 5]. Owing to intra- and intertumor genetic heterogeneity, single-site tissue biopsy may not represent the full-scale tumor genomic characterization in real time. Tissue biopsy also has limitations in screening for early-stage cancer. It is imperative to explore a substitute or complementary actionable approach to grasp the genetic diversity of tumor tissue.

ctDNA, derived from necrotic and apoptotic tumor cells of multiple lesions, has become a promising biomarker. It may provide the whole scale of tumor genomic profiles with the advantages of being noninvasive. Considering the short half-life of ctDNA, it is also expected to be a sensitive marker to reflect tumor status and treatment efficacy in real time. But, the concentration of ctDNA in the cell-free DNA (cfDNA) varied, ranging from less than 0.01% to more than 90% [6]. It was found to be correlated with tumor bulk, stage [7], and types [8]. ctDNAs could be detected in metastatic cancers more easily than in localized ones ([8]). The level of ctDNA in malignant BC patients was higher than in nonmalignant and healthy controls independent of clinicopathological parameters [9]. Thus, it is more difficult to detect ctDNA in early stage and localized tumors with relatively small volume, which may limit the clinical applications of ctDNA. Investigating the concordance of mutations between plasma and tissue could help to evaluate the possibility of clinical application of NGS-based liquid biopsy.

Accumulating studies have investigated the concordance between two biopsies in various cancers, with varied concordance rate. Previous studies in BC found that 15.6% to 48% tissue single nucleotide variants (SNVs) and insertions and deletions (InDels) could be detected in ctDNA, and 33% to 48% ctDNA SNVs and InDels could be detected in tissues in studies with the sample size of 62 and 45, respectively [10, 11]. As for gene amplification, amplifications of *FGFR1*, *c-MYC*, *MDM2*, and *CDK4* detected by whole-exome sequencing in a BC tissue were also found in ctDNA by targeted sequencing [12]. Overexpression of *ERBB2* is correlated with poor prognosis and reduced survival in breast tumors. *HER2* antagonists were approved and widely adopted for the treatment of *HER2*-overexpressed BC. Liang et al. reported a high agreement of *ERBB2* amplification between tissues and plasmas with Cohen's kappa of 0.77, compared with the low agreement of *EGFR* amplification with Cohen's kappa of 0.33 [13]. In this study, we evaluated the concordance rate of different kinds of genomic alterations between paired tissue biopsies and ctDNA samples in a Chinese BC cohort.

## 2. Materials and Methods

### 2.1. Study Design and Patient Sample Collection

Eighty-one patients diagnosed as BC were included in this study. Clinical TNM classification was defined according to American Joint Committee on Cancer (Breast Cancer Staging, 7th Edition). Tumor tissues and blood samples were collected from 81 patients. The tumor tissue samples were collected by either percutaneous needle biopsy or surgery. The blood samples were collected in Streck tubes (La Vista, Nebraska). For concordance study, the blood samples were collected at least one day before surgery.

This study was approved by the Ethics Committee of Cancer Hospital, Chinese Academy of Medical Sciences, and conformed to the provisions of the Declaration of Helsinki. Every patient signed an informed consent form. Patients were selected according to the following criteria: (1) age of 18 or above; (2) those who fulfilled the diagnostic criteria of NCCN Clinical Practice Guidelines in Oncology for BC. Patients were excluded according to following criteria: (1) who had second primary tumor before enrollment; (2) who had poor control of medical treatment with severe cardiovascular, cerebrovascular diseases, or diabetes; (3) noncompliant, or there was a situation that researchers thought it was not suitable to be included in the study; (4) pregnant or lactating women; (5) who had previous transplant surgery; (6) who had previous stem cell treatment; (7) who had received allogeneic blood transfusion within one year and immunotherapy within four weeks.

### 2.2. Sample Processing and DNA Extraction

Whole-blood samples were processed within three days after blood collection, and they were separated into plasma and buffy coat by two-step centrifugations. Tissues, plasma, and white blood cell (WBC) samples were stored at −80°C until further processing. Circulating cell-free DNA (cfDNA) was extracted from 3-4 mL plasma by the MagMAX™ Cell-Free DNA Isolation kit (Life technologies, A29319, USA). Genomic DNAs (gDNAs) were extracted from tumor tissue and buffy coat using a Qiamp FFPE tissue kit (Qiagen, 56404, Germany) and TiANamp Blood DNA Maxi kit (TianGen Biotech, DP332, China), respectively. Qubit dsDNA high-sensitivity (HS) assay kit (Invitrogen, Q32854, USA) was used to quantify the DNA concentration.

### 2.3. Library Preparation, Target Region Capture, and Sequencing

gDNAs from tumor tissue and buffy coat were sheared into 150–250 bp fragments by Covaris S220 (Covaris, S220, USA). The cfDNA and gDNA libraries were prepared by KAPA Hyper Prep Kit (KAPA, KK8504, USA) according to the manufacturers' instructions. One to five libraries from the same sample type were pooled at an equal molar concentration and hybridized to a customized Agilent SureSelect panel (∼0.45 M). After PCR amplification, the quality and quantity of captured library were assessed by an Agilent 2100 bioanalyzer and ABI 7500 real-time PCR system (Life Technologies, 4351107, USA). Sequencing libraries were loaded on a Nextseq 500 (Illumina, San Diego, CA) sequencing platform to generate 75 bp pair-end reads. The minimal read depths of plasma, tissue, and buffy coat were 800x, 800x, and 250x, respectively.

### 2.4. Bioinformatics Analysis

FASTQ files were generated by bcl2fastq2 (v.2.17.1.14). The raw reads containing P5/P7 adapters were trimmed off, and the reads containing over 30% low-quality (base quality <30) sequencing bases and over 60% N bases were also discarded. Then, the clean reads were mapped to the GRCh37/hg19 human genome using Burrows-Wheeler Aligner (BWA, v.0.7.12, http://bio-bwa.sourceforge.net/) MEM algorithm. PCR duplicates were marked using Picard tools (v.1.119, https://broadinstitute.github.io/picard/). Paired sample mutations, including SNVs and InDels, were called by paired-mode of VarScan (v.2.4.3, http://varscan.sourceforge.net/index.html) with verified settings by comparing ctDNAs or tissue DNAs to matched germline control buffy coat DNAs. CNVkit (v.0.8.6, https://cnvkit.readthedocs.io/en/stable/) was used to identify copy number variations (CNVs). Functional annotations of somatic SNVs and InDels were performed using ANNOVAR (2015 Jun 16, http://annovar.openbioinformatics.org/en/latest/). Candidate mutations were filtered if the (1) depth was below 800x for tissues and ctDNAs and reads supporting alternative allele were below 4; (2) reads with strand bias; (3) variant allele frequency (VAF) < 1% and <0.5% for tissues and ctDNAs; (3) variations were synonymous, with unknown significance and in introns; (4) mutations were not recorded in the COSMIC database (v89); (5) VarScan marked as SS = 0, SS = 1, SS = 3, or SS = 5.

### 2.5. Statistical Analysis

Concordance of SNVs and InDels was defined as that the same mutation could be found in both tissue and plasma. Discordance was defined as mutation could be only detected in one biopsy. The concordance for gene amplification was defined as the gene amplification found or absent in both biopsies.

The continuous normally distributed variables were represented using mean and standard deviation (SD), and the continuous abnormally distributed variables were represented using median and interquartile range (IQR) and counting number and percentage for categorical variables. Mutation density was calculated as mutation counts within the gene divided by the length of the coding region of the gene. For the comparison of VAF levels, we applied the Wilcoxon rank sum test with a statistically significant level of *p* < 0.05. Tumor tissue biopsy sequencing was used as a gold standard to evaluate the performance of ctDNA. True positive (TP), false positive (FP), false negative (FN), and true negative (TN) were the number of alterations detected in two biopsies, in ctDNAs only, absent from ctDNAs only, and absent from two biopsies, respectively. We calculated the sensitivity (TP/(TP + FN)), specificity (TN/(TN + FP)), positive predictive value (PPV) (TP/(TP + FP)), negative predictive value (NPV) (TN/(TN + FN)), and diagnostic accuracy ((TP + TN)/(TP + TN + FP + FN)) value. All the statistical analysis and graphics used *R* (v.3.4.0, https://www.r-project.org/).

## 3. Results

### 3.1. Panel Design and Quality Control

The clinical characteristics of BC patients included in this study are summarized in Supplementary Table 1. All BC patients were women. The mean age of all the patients was 53.3 ± 12.1, ranging from 28–90. Seven (7/81, 8.6%) patients were younger than 40 years, 49 (49/81, 60.5%) patients were between 40 and 60 years, and 25 (25/81, 30.9%) patients were older than 60 years. Patients with stages I, II, III, and IV accounted for 3.7% (3/81), 27.2% (22/81), 53.1% (43/81), and 4.9% (4/81), respectively. And, for the 41 patients with preoperative blood, the median age was 53.0 (IQR 17.0), ranging from 39 to 75. Patients with stages I, II, III, and IV accounted for 7.0% (3/41), 31.7% (13/41), 46.3% (19/41), and 9.8% (4/41), respectively.

The custom-designed 136-gene-panel (Agilent) used in this study (Supplementary Table 2) covered 482 kb of the human genome. The selection criteria for target region were based on the data of BC in the Cancer Genome Atlas (TCGA, https://www.cancer.gov) and several BC-related researches [14–16]. The captured regions included the important driver genes associated with BC and other solid tumors and targets related with targeted therapy, chemotherapy, and immunotherapy of BC. The panel contained the entire coding regions and partial introns of the genes, which ensured the detections of both mutations and CNVs. The average depths were 1435x (range 824x–3157x), 1752x (range 976x–3658x), and 660x (range 356x–1072x) for tissues, ctDNAs, and buffy coat, respectively. The mean on-target fractions were 61.0% and 55.8% for tissues and ctDNAs. And, the mean fractions of target region coverage were 100.00% (range 99.89%–100.00%) and 99.99% (range 99.96–100.00%) for tissues and ctDNAs.

### 3.2. Overall DNA Alteration Profile

**Figure 1:**
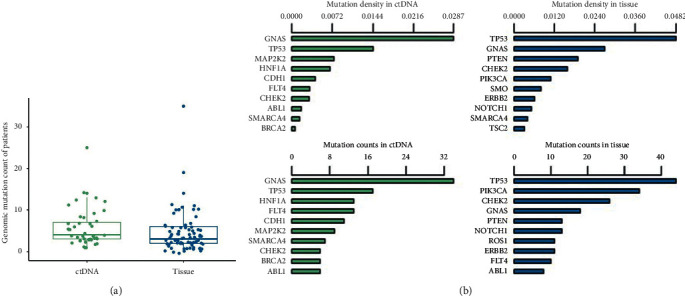
Comparison of the mutation number in different biopsies. (a) Mutation counts in 41 ctDNA samples and 81 tissue samples. One dot in the figure represented as the mutation counts of one sample. (b) Mutation counts and density of the top 10 genes in two biopsies. Green and blue bars represented the mutation counts or density in ctDNAs and tissues, respectively.

In total, 387 mutations were detected in 76 (93.8%, 76/81) BC tissue samples, including 350 SNVs and 37 InDels. The median genomic mutations per sample in tissues were 3.0 (IQR 4.0) with mean of 4.8 (SD 5.5) (Figure 1(a)). The median genomic mutations per sample in early- and late-stage BC samples were 4.0 (IQR 7.0) and 3.0 (IQR 4.0) with mean of 5.7 (SD 7.2) and 4.4 (SD 4.9), respectively. In ctDNAs, we detected 239 mutations in 41 samples, including 140 SNVs and 99 InDels. The median mutation counts per sample were 4.0 (IQR 4.0) with mean of 5.8 (SD 4.6) (Figure 1(a)). The median genomic mutations per sample in early- and late-stage ctDNA sample were 4.0 (IQR 2.3) and 5.0 (IQR 5.0) with mean 4.8 (SD 3.4) and 6.0 (SD 3.5), respectively. There was no difference in mutation counts between early- and late-stage samples in two biopsies (*p* > 0.05; Wilcoxon rank sum test). And, the average mutations per sample in ctDNAs were greater than in tissues (*p*=0.0254; Wilcoxon rank sum test), with 65.9% (27/41) ctDNA samples harboring more mutations than tissue samples.

Then, we investigated the mutation level among targeted genes. *TP53*, *NOTCH1*, *PIK3CA*, *GNAS*, and *CHEK2* were the top five genes carrying most mutations in tissues and with the mutation counts in 81 samples of 44, 34, 26, 18, and 13, respectively. And, the top five genes with the highest mutation density in tissues were *TP53*, *GNAS*, *PTEN*, *CHEK2*, and *PIK3CA* (Figure 1(b)). As for ctDNAs, *GNAS*, *TP53*, *HNF1A*, *FLT4*, and *CDH1* were the top five most frequently mutated genes in 41 ctDNA samples, and the mutation counts for these genes were 34, 17, 9, 13, and 11, respectively. *TP53* and *GNAS* were the most frequently mutated genes in two biopsies. And, the top five genes with the highest mutation density in ctDNAs were *GNAS*, *TP53*, *MAP2K2*, *HNF1A*, and *CDH1* (Figure 1(b)). Among the top mutated genes, *TP53*, *PIK3CA*, and *CDH1* were also the top three mutated genes in 64258 BC cases across 12 projects in TCGA database.

As for gene amplification, 43 amplifications of 12 genes were identified in two biopsies of 81 tissue samples and 41 plasma samples. The affected genes included *EGFR, ERBB2, FGFR1, FGFR2, GNAS, IGF1R, JAK2, JAK3, MYC, NCCRP1, NTRK3*, and *ROS1.* 76.7% (33/43) were found in tissues, and 23.3% (10/43) were found in ctDNAs only. The most frequent amplified gene was *ERBB2*, whose amplification was detected in 18 patients, including 17 in tissues and three in ctDNAs. The number of genomic alterations in detected genes in two biopsies is summarized in Supplementary Figure 1.

### 3.3. Concordance between Tissue Biopsy and ctDNA

**Figure 2:**
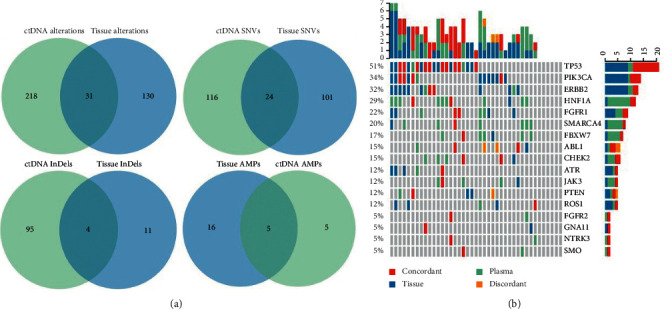
Concordant and discordant alterations detected in ctDNAs and tissues. (a) Venn diagrams representing the counting number of alterations, SNVs, InDels, and detected amplifications (AMPs) detected only in tissues (blue), detected only in ctDNAs (green), and detected in both biopsies (overlap). (b) Oncoprint chart for 17 genes which had at least one concordant protein-coding mutation site or concordant gene amplification across all 41 patients. One vertical bar represents one patient. Green bar: mutations in plasma; blue bar: mutations in tissue; red bar: concordant mutation in two biopsies; orange bar: mutations in the same gene, but discordant in ctDNA and tissue.

For 41 patients with paired tissue and liquid biopsy samples, a total of 410 alterations were detected in two biopsies, including 161 alterations in tissues and 249 alterations in ctDNAs. 31 alterations were shared by both biopsies, including 24 SNVs, four InDels, and five amplifications (Figure 2(a)). For protein-coding mutation level, 16 (16/41, 39.0%) patients had at least one concordant mutation. The concordance rate for detected mutations ranged from 0.0% to 60.0% across all patients. 20.0% (28/140) tissue-derived mutations could be detected in ctDNAs, and 11.7% (28/239) ctDNA-derived mutations could be found in paired tissues.

At the gene level, the concordance rate for each patient, including genes with detected mutation and mutation absent from both biopsies, ranged from 83.8% (114/136) to 99.3% (135/136). The concordance of 41 paired samples with detected mutations at gene level ranged from 0.0% (0/41) to 24.4% (10/41). The top genes with the highest concordance rate (Figure 2(b)), proto-oncogenes like *TP53* or tumor suppressor genes like *ERBB2* and *PIK3CA*, were highly mutated in various cancers.

For gene amplifications, five patients were detected with gene CNVs in both biopsies, and gene amplification were absent from 23 patients. The overall concordance rate was 68.3%. Given BC patients with *ERBB2* amplification had a clinical benefit from trastuzumab, we evaluate the concordance rate for *ERBB2* amplification with the concordant value of 85.4% (Table 1), including two patients with concordant *ERBB2* amplification and 33 patients with no detectable *ERBB2* amplification. Also, we calculated Cohen's Kappa value which was 0.332.

### 3.4. Comparison of VAF between Concordant and Discordant Mutations in ctDNAs

**Figure 3:**
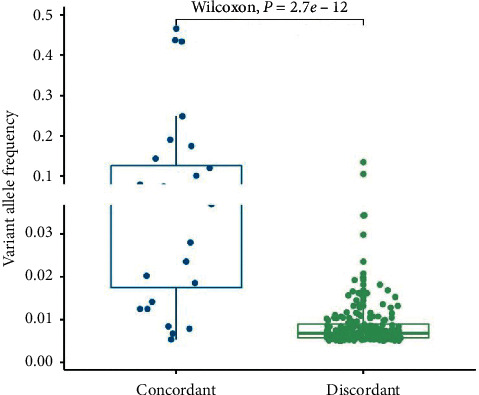
Comparison of VAF between concordant and discordant ctDNA mutations. Boxplot displayed the minimum, maximum, median, and interquartile range of VAF of ctDNA. Concordant ctDNA mutations had higher frequency than discordant ctDNA mutations (*p* < 0.001, Wilcoxon rank sum test).

We compared the VAF of concordant and discordant somatic mutations in ctDNA (Figure 3). The overall median VAF was 0.7% (range 0.5%–46.6%) with a mean of 2.1% (SD 5.6%). The median level of VAF for concordant ctDNA mutations (6.6%) was higher than that for discordant ones (0.7%) (*p* < 0.001; Wilcoxon rank sum test). 85.7% concordant protein-coding mutations had VAF higher than 1%. For discordant mutations, 78.7% mutations had VAF lower than 1%, which may be a result of tumor heterogeneity and could only be detected in plasma but not single-site tissue biopsy.

### 3.5. Evaluation of Concordance Performance Analysis

We analyzed the sensitivity, specificity, PPV, NPV, and diagnostic accuracy across five genes which carried the most concordant mutations, including *TP53, PIK3CA, CHEK2, HNF1A*, and *ABL1* (Table 2) with Cohen's Kappa values of 0.271, 0.254, 0.386, 0.288, and 0.549, respectively. Tumor tissue biopsy sequencing was used as a gold standard. The combined sensitivity and specificity of the five genes were 49.0% and 85.9%, respectively, and the diagnostic accuracy was 76.6%. At the gene level, single gene sensitivity and specificity ranged from 28.6%–66.7% and 74.1%–94.6%, respectively. *CHEK2* and *HNF1A* showed overall high sensitivity and specificity. The sensitivity and specificity of *ERBB2* amplification were 28.6% and 97.1%, respectively (Table 1).

### 3.6. Concordance between Tissue Biopsy and ctDNA in Other Cancer Types

We selected six liver cancer and 20 colorectal cancer patients and evaluated the concordance between ctDNA and tissue biopsy. There were 13 females and 13 males. And, the mean age was 60.0 (SD 12.0) with the median of 60.0 (IQR 15.0). Patients with stages I, II, III, and IV accounted for 0.0% (0/26), 11.5% (3/26), 46.2% (12/26), and 34.6% (9/26), respectively (Supplementary Table 3).

A total of 119 alterations were detected in tissues, including 90 SNVs, 20 InDels, and 9 CNVs, and 189 alterations in ctDNAs, including 140 SNVs, 46 InDels and 3 CNVs. The median genomic mutations per sample were 4.0 (IQR 3.0) with mean of 4.0 (SD 2.4) in tissues, and 4.0 (IQR 3.8) with mean of 7.2 (SD 10.1) in ctDNAs. There was no difference of the average mutations per sample between tissue and ctDNA (*p*=0.7257, Wilcoxon rank sum test). The top five genes carried most mutations were *APC*, *TP53*, *KRAS*, *SMAD4*, and *GNAS* with the mutation counts of 32, 20, 14, 4, and 3 in tissues and *GNAS*, *APC*, *TP53*, *FLT4*, and *GATA6* with mutation counts of 19, 12, 10, 9, and 9 in ctDNAs.

For the protein-coding mutation level, 15 SNVs and five InDels were detected in two biopsies. 46.2% (12/26) samples had at least one concordant mutation in two biopsies; in the meantime, the concordance rate ranged from 0.0% to 50.0% across 26 samples. 18.2% (20/110) tissue-derived mutations were detected in ctDNAs, and 10.8% (20/186) ctDNA-derived mutations were detected in tissues. For gene amplifications, two patients had concordant CNVs and 20 patients had no gene amplifications in two biopsies. The overall concordance rate was 84.6% (22/26).

## 4. Discussion

Tumor heterogeneity has been widely observed, which may lead to sampling bias and loss of valuable clinical information. Liquid biopsy like ctDNA analysis was expected to overcome the genomic heterogeneity within tumor. But, related studies in BC are still limited compared with those in lung and gastrointestinal cancer. To investigate the concordance of genomic alterations, we used paired samples with normal controls in this study. Previous studies in American and Japanese patients with BC showed that the concordance rates ranged from 10.8% to 74.3%. According to Chea's study, 10.8% concordant mutations were detected in both biopsies among 45 American patients [11]. Another study reported a higher concordance rate in 38 advanced BC patients from America [10], which found that 48% (32/67) tissue mutations were detected in ctDNAs and 48% (35/72) ctDNA mutations were found in tissues. Few studies were conducted in East Asia cohorts. In Chinese patients with BC, a previous study reported 80% (4/5) concordant rate in five metastatic BC patients [17]. Recently, Zhou [18] evaluated the concordance among 48 primary BC patients. The results showed that 94 (61.03%) of 154 tissue mutations could be detected in paired ctDNAs, and 60 mutations were exclusive. Here, we showed that 17 of 41 patients had at least one concordant alteration and 33 of 41 patients had at least one concordant alteration plus undetected amplification. 20.0% (28/140) tissue-derived mutations could be detected via ctDNA-based sequencing. 11.7% (28/239) ctDNA-derived mutations could be found in paired tissues. We also evaluated the concordance between the two biopsies in 26 liver cancer and colorectal cancer patients, and the concordance rate was similar. The relatively low concordance rate may be caused by the high heterogeneity of breast tumor on one hand and the low ctDNA fraction in plasma on the other hand [6]. Another study [19] reported that 53.2% ctDNA mutations were consistent with clonal hematopoiesis, which may influence the concordance between tissue and plasma because the mutations were absent in tissues. To sum up, the variation in the concordant rate among different studies might arise from the differences in sample collection, technology, and definition of concordance.

Moreover, VAFs of concordant ctDNA mutations were much higher than those of discordant ones in our study, which was consistent with the previous study [11]. This indicated that ctDNA collected the genomic information from multiple tumor clones and captured tumor heterogeneity. These added evidence to the idea that ctDNA analysis could serve as a complementary tool to traditional biopsy by providing complementary information on genetic characterizations.

It is important to note that we found a relatively high concordant rate of *ERBB2* amplification. Amplification of *ERBB2* has been proven to be strongly associated with the development of BC and poor prognosis [20]. BC patients with *ERBB2* amplification could benefit from trastuzumab [21–24]. But, immunohistochemistry (IHC) and fluorescence in situ hybridization (FISH) [25, 26] often yield discordant *ERBB2* status, which required multiple tissue samples to achieve robust clinical results. The concordance rate of *ERBB2* amplification in our study was 85.4%, which indicated that ctDNA could serve as a useful indicator to assess *ERBB2* amplification state in breast tumor.

Overall, our findings contributed to the understanding of tumor heterogeneity and provide more information on the clinical value of ctDNA. Precision medicine on solid tumors based on the genomic alteration profiles is promising and effective [27]. Given the limited overlapped mutations between ctDNA and tissue, it may be necessary to integrate the liquid biopsy with the tissue biopsy which may provide more informative evidence on operational targeted therapy. It would be a plus to reveal the gene mutation difference from the experimental aspect like sample processing procedure in the future study. Besides, a large cohort study will be needed to validate the results and evaluate to what extent plasma ctDNA could overcome tumor heterogeneity.

## 5. Conclusions

Our study evaluated the concordance of genomic alterations between solid and liquid biopsies in 41 breast tumor patients. 39.0% patients had at least one concordant mutation in two biopsies. 20.0% tissue-derived mutations could be detected via ctDNA-based sequencing, whereas 11.7% ctDNA-derived mutations could be found in paired tissues. VAF was higher in concordant ctDNA mutations than that in the discordant ones. For gene amplifications, *ERBB2* had a high concordance rate of 85.4%, suggesting that ctDNA may become a useful tool to monitor therapeutic process targeting *ERBB2* amplification. These findings provided more information about ctDNA becoming a complementary tool of tissue biopsy of breast cancer in a noninvasive manner.

## Figures and Tables

**Table 1:** Concordance of *ERBB2* amplification for breast cancer.

		Tissues		Sensitivity	Specificity	Concordance
		(+)	(−)	(%)	(%)	(%)
ctDNA	(+)	2	1			
	(−)	5	33	28.6	97.1	85.4

**Table 2:** Sensitivity, specificity, and diagnostic accuracy across 6 genes.

ctDNA mutations	Tissue mutations		Sensitivity	Specificity	PPV	NPV	Diagnostic	Youden's
	(+)	(−)	(%)	(%)	(%)	(%)	Accuracy (%)	J Index
*TP53*								
(+)	10	7						
(−)	9	20	52.6	74.1	58.8	70.0	65.2	0.27

*PIK3CA*								
(+)	4	2						
(−)	10	27	28.6	93.1	66.7	73.0	72.1	0.22

*CHEK2*								
(+)	2	4						
(−)	1	35	66.7	89.7	33.3	97.2	88.1	0.56

*HNF1A*								
(+)	4	9						
(−)	2	29	66.7	76.3	30.8	93.5	75.0	0.43

*ABL1*								
(+)	4	2						
(−)	3	35	57.1	94.6	66.7	92.1	60.9	0.52

Total positive	24	24						
Total negative	25	146						
Total(positive + negative)	49	170	49.0	85.9	50.0	85.4	77.6	0.35

## Data Availability

The analyzed data sets used to support the findings of this study are available from the corresponding author upon request.

## Supplementary Materials

Supplementary Materials Figure S1: the number of genomic alterations in detected genes of two biopsies. Table S1: clinical characteristics of all BC patients; Table S2: genes included in the panel; and Table S3: clinical characteristics of liver cancer and colorectal cancer patients.
