# Supplementary material for: Concordance of Genomic Alterations between Circulating Tumor DNA and Matched Tumor Tissue in Chinese Patients with Breast Cancer
Source: J Oncol. 2020 Aug 27;2020:4259293. doi: 10.1155/2020/4259293 (PMC7474381; doi:10.1155/2020/4259293)
Supplement: Supplementary Materials — Figure S1: the number of genomic alterations in detected genes of two biopsies. Table S1: clinical characteristics of all BC patients; Table S2: genes included in the panel; and Table S3: clinical characteristics of liver cancer and colorectal cancer patients. [file 4259293.f1.zip › 4259293.f1/Supplementary_Figure_1.pdf]

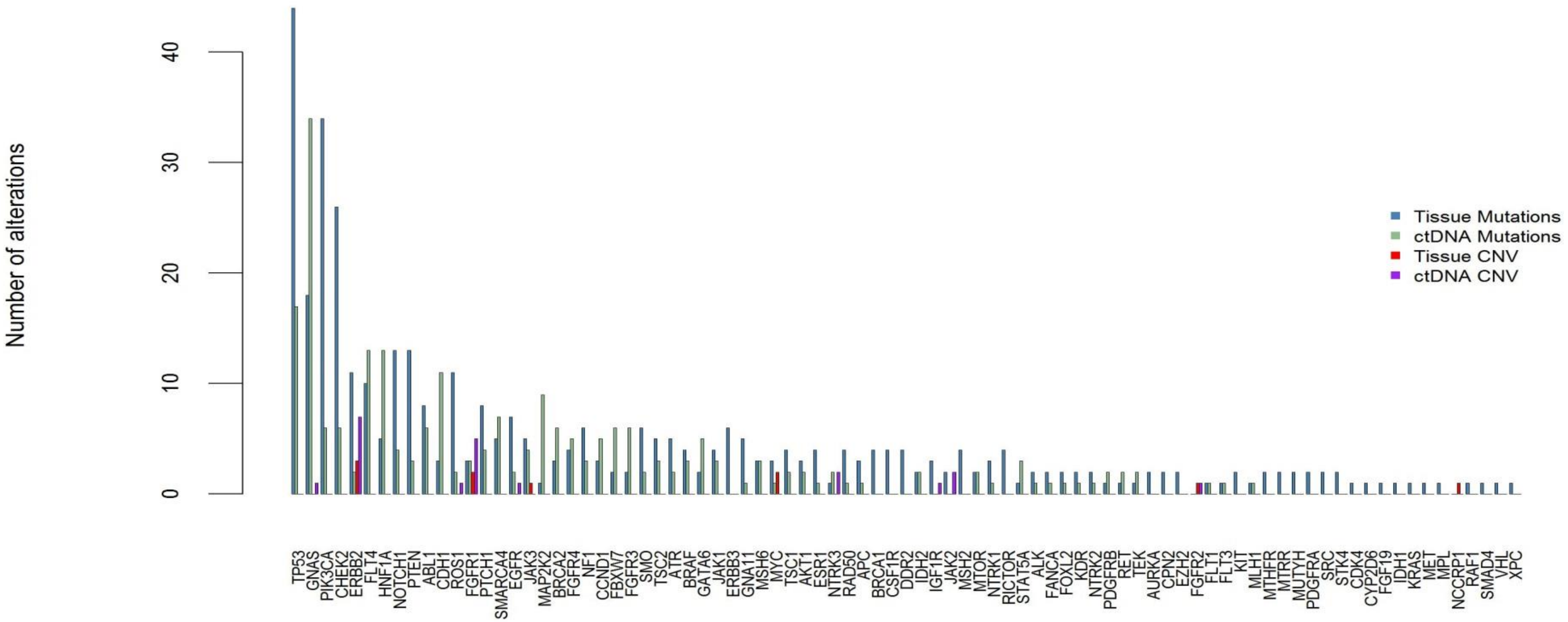

Supplemental Fig. 1

Mutation and amplification counts at the gene level. Blue and green bars represent the mutation counts of genes in tissues and ctDNAs. Red and purple bars represent the gene amplification in tissues and ctDNAs.
