# Supplementary material for: Concordance of Genomic Alterations between Circulating Tumor DNA and Matched Tumor Tissue in Chinese Patients with Breast Cancer
Source: J Oncol. 2020 Aug 27;2020:4259293. doi: 10.1155/2020/4259293 (PMC7474381; doi:10.1155/2020/4259293)
Supplement: Supplementary Materials — Figure S1: the number of genomic alterations in detected genes of two biopsies. Table S1: clinical characteristics of all BC patients; Table S2: genes included in the panel; and Table S3: clinical characteristics of liver cancer and colorectal cancer patients. [file 4259293.f1.zip › 4259293.f1/Supplementary_Table_1.pdf]

Table S1 Clinical characteristics of patients

Patients with tissue biopsies(n=84)	Number	Percentage	Patients with liquid biopsies(n=41)	Number	Percentage
Age(years)			Age(years)		
Mean ±SD	53.6 ± 12.2		Median(IQR)	53.0 (17.0)	
Stage			Stage		
Ⅰ	3	3.6%	Ⅰ	3	7.0%
Ⅱ	25	29.8%	Ⅱ	13	31.7%
Ⅲ	43	51.2%	Ⅲ	19	46.3%
Ⅳ	4	4.8%	Ⅳ	4	9.8%
NA	9	10.7%	NA	2	4.9%
