# Supplementary material for: Concordance of Genomic Alterations between Circulating Tumor DNA and Matched Tumor Tissue in Chinese Patients with Breast Cancer
Source: J Oncol. 2020 Aug 27;2020:4259293. doi: 10.1155/2020/4259293 (PMC7474381; doi:10.1155/2020/4259293)
Supplement: Supplementary Materials — Figure S1: the number of genomic alterations in detected genes of two biopsies. Table S1: clinical characteristics of all BC patients; Table S2: genes included in the panel; and Table S3: clinical characteristics of liver cancer and colorectal cancer patients. [file 4259293.f1.zip › 4259293.f1/Supplementary_Table_2.pdf]

Table S2 Genes included in the panel

ABCB1	*ABL1*	*AKT1*	*ALK*	*APC*	*ATIC*	*ATM*	*ATR*
AURKA	*BRAF*	*BRCA1*	*BRCA2*	*CBR3*	*CCND1*	*CD3EAP*	*CDH1*
CDK1	*CDK4*	*CDK6*	*CDKN2A*	*CHEK2*	*CPN2*	*CSF1R*	*CTNNB1*
CYP1B1	*CYP2D6*	*DDR2*	*DPYD*	*EGFR*	*ERBB2*	*ERBB3*	*ERBB4*
ERCC1	*ESR1*	*EZH2*	*FANCA*	*FBXO11*	*FBXW7*	*FCGR3A*	*FCGR3B*
FGF19	*FGFR1*	*FGFR2*	*FGFR3*	*FGFR4*	*FLT1*	*FLT3*	*FLT4*
FOXL2	*GATA6*	*GNA11*	*GNAQ*	*GNAS*	*GNG7*	*GSTP1*	*HAS3*
HECW1	*HIF1A*	*HNF1A*	*HRAS*	*IDH1*	*IDH2*	*IGF1R*	*INSRR*
JAK1	*JAK2*	*JAK3*	*KDR*	*KIT*	*KRAS*	*LRCH1*	*LRRFIP2*
MAP2K1	*MAP2K2*	*MET*	*MLH1*	*MPL*	*MSH2*	*MSH6*	*MTHFR*
MTOR	*MTRR*	*MUTYH*	*MYC*	*MYD88*	*NCCRP1*	*NF1*	*NOTCH1*
NPM1	*NQO1*	*NRAS*	*NT5C2*	*NTRK1*	*NTRK2*	*NTRK3*	*NUDT15*
PALM2	*PCLO*	*PDGFRA*	*PDGFRB*	*PIK3CA*	*PTCH1*	*PTEN*	*RAD50*
RAF1	*RB1*	*RET*	*RICTOR*	*ROS1*	*SDK1*	*SEMA3C*	*SH2D2A*
SMAD4	*SMARCA4*	*SMO*	*SNAPC5*	*SOD2*	*SRC*	*SRP19*	*STAT5A*
STK11	*STK4*	*SYNE1*	*TEK*	*TOE1*	*TP53*	*TPMT*	*TSC1*
TSC2	*UMPS*	*UST*	*VHL*	*XPC*	*XRCC1*	*ZDHHC14*	*ZNF276*
