# Supplementary material for: Concordance of Genomic Alterations between Circulating Tumor DNA and Matched Tumor Tissue in Chinese Patients with Breast Cancer
Source: J Oncol. 2020 Aug 27;2020:4259293. doi: 10.1155/2020/4259293 (PMC7474381; doi:10.1155/2020/4259293)
Supplement: Supplementary Materials — Figure S1: the number of genomic alterations in detected genes of two biopsies. Table S1: clinical characteristics of all BC patients; Table S2: genes included in the panel; and Table S3: clinical characteristics of liver cancer and colorectal cancer patients. [file 4259293.f1.zip › 4259293.f1/Supplementary_Table_3.pdf]

Table S3 Clinical characteristics of other cancers

Patients(n=26)	Number	Percentage
Cancer		
Liver cancer	6	23.1%
Colorectal cancer	20	76.9%
Age(Years)		
Mean ±SD	60.0 ±12.0	
Gender		
Male	13	50.0%
Stage		
Ⅰ	0	0.0%
Ⅱ	3	11.5%
Ⅲ	12	46.2%
Ⅳ	9	34.6%
NA	2	7.7%
